# SARS in Teaching Hospital, Taiwan

**DOI:** 10.3201/eid1010.040356

**Published:** 2004-10

**Authors:** Yee-Chun Chen, Ming-Fong Chen, Shuen-Zen Liu, James C. Romeis, Yuan-Teh Lee

**Affiliations:** * National Taiwan University Hospital, Taipei, Taiwan;; † National Taiwan University College of Medicine, Taipei, Taiwan;; ‡ National Taiwan University College of Management, Taipei, Taiwan;; § Saint Louis University, St. Louis, Missouri, USA

**Keywords:** letter, SARS, hospital management

**To the Editor:** During the global epidemic of severe acute respiratory syndrome (SARS), the illness was transmitted rapidly within hospitals, which created pools of persons who became infected and through whom the disease was spread. Intrahospital transmission amplified regional outbreaks and augmented spread of the illness into the community (*1**–**3*). Healthcare workers, hospital patients, and hospital visitors accounted for 18%–58% of all cases of SARS in the five countries with the largest outbreaks (*1**,**2*). The concentration of SARS among hospital staff strained hospital facilities, personnel, and finances.

National Taiwan University Hospital, established more than 100 years ago, is the first teaching hospital and the best resource in Taiwan for managing patients with illnesses that are difficult to treat. The hospital has 2,400 beds and provides primary and tertiary care services in Taipei. Taipei City was among the hardest hit areas by SARS in the world (*3*). From March 10 to July 23, 2003, the hospital reported 270 patients with SARS, many of whom were severely ill. The hospital treated 180 of the 665 patients with SARS reported in Taiwan, even though it was staffed by 4,450 of the country's 178,000 healthcare workers. SARS had an impact on this hospital for three likely reasons. First, the hospital identified and treated the first SARS patients in Taiwan (*4**,**5*). Second, it provided easy access through the emergency room and outpatient clinics. Febrile persons with a travel history to SARS-affected areas or other risk of SARS exposure came directly to this hospital for care. Third, many hospitals, particularly private facilities, were reluctant to report and admit patients with SARS during the early stage of the epidemic because of financial considerations and fear.

The hospital felt the brunt of the epidemic in Taiwan during early May 2003, which paralleled the severity of the SARS epidemic in Taipei (*3**,**4**,**6*). The maximal number of SARS patients admitted to the hospital within 24 hours was 12 on May 3. The maximal number of SARS patients reported within 24 hours was 15 on May 6; 8 patients were transferred to other hospitals on May 7. However, 18 patients stayed overnight in the emergency room on May 7. Subsequently, SARS developed in 12 emergency room healthcare workers (*6**,**7*).

Our preliminary studies showed that the average inpatient cost for patients with SARS was not higher than for patients with pneumonia, after adjustment for age, sex, and length of stay (MF Chen, unpub. data). However, SARS caused financial and operational disruptions in the hospital. During this period, hospital utilization rates decreased. Compared with the previous year's rates, outpatient and emergency visits fell to 37%, inpatient admissions fell to 29%, and surgical procedures fell to 15%. Bed occupancy decreased from 86% in May 2002 to 38% in May 2003. SARS also imposed physical and psychological concerns on the healthcare workers.

During the later stage of the SARS epidemic, the Taiwan government offered special financial assistance to hospitals and healthcare workers as an incentive to help fight SARS. The country's National Health Insurance program compensated hospitals for the decrease in revenues, based on the hospital's reimbursement amount before the SARS epidemic. This measure was effective in motivating other hospitals to accept patients with SARS. The proportion of inpatients with SARS at the hospital dropped from 79.5% during March 10 to April 23, to 46.2% during April 24 to May 1, to 11.6% during May 2 to July 23. This financial assistance program remarkably reduced the impact on the hospital as other hospitals began treating patients with SARS.

Preparations for a medical emergency must address the availability and quality of medical care as well as the implications for public health policy, including political, legal, social, financial, and ethical issues (*1*). The importance of a sound financial policy cannot be overemphasized. Since the 1980s, healthcare systems have become free market enterprises. Laws and regulations are needed to allow governments to mobilize the resources of all hospitals and compensate them during health crises. Government agencies need to work together with the healthcare system, including health insurance systems and social services, well in advance of epidemic emergencies to maximize limited resources and distribute them equitably.

Democratic societies must preserve human rights (including the right to medical care and freedom from fear), while respecting and protecting the rights and safety of hospitals and healthcare workers. We now face the potential resurgence of SARS, other emerging and reemerging infectious diseases, and the threat of bioterrorism. Careful consideration of the financial issues of hospital management should be an important part of social policy. The emergence of SARS provides a reminder of the potential threat to the entire healthcare system when a new disease suddenly appears. A major lesson from the SARS experience is that government planning and intervention are required.
